# Top Common Differentially Expressed Genes in the Epileptogenic Nucleus of Two Strains of Rodents Susceptible to Audiogenic Seizures: WAR and GASH/Sal

**DOI:** 10.3389/fneur.2020.00033

**Published:** 2020-02-13

**Authors:** Samara Damasceno, Ricardo Gómez-Nieto, Norberto Garcia-Cairasco, Manuel Javier Herrero-Turrión, Faustino Marín, Dolores E. Lopéz

**Affiliations:** ^1^Institute of Neurosciences of Castilla y León, University of Salamanca, Salamanca, Spain; ^2^Salamanca Institute for Biomedical Research, Salamanca, Spain; ^3^Department of Physiology, University of São Paulo, Ribeirao Preto, Brazil; ^4^INCYL Neurological Tissue Bank (BTN-INCYL), Salamanca, Spain; ^5^Department of Human Anatomy and Psychobiology, School of Medicine, University of Murcia, Murcia, Spain

**Keywords:** audiogenic model, microarray, RNA-Seq, seizure, transcriptome

## Abstract

The Wistar Audiogenic Rat (WAR) and the Genetic Audiogenic Seizure Hamster from Salamanca (GASH/Sal) strains are audiogenic epilepsy models, in which seizures are triggered by acoustic stimulation. These strains were developed by selective reproduction and have a genetic background with minimal or no variation. In the current study, we evaluated the transcriptome of the inferior colliculus, the epileptogenic nucleus, of both audiogenic models, in order to get insights into common molecular aspects associated to their epileptic phenotype. Based on GASH/Sal RNA-Seq and WAR microarray data, we performed a comparative analysis that includes selection and functional annotation of differentially regulated genes in each model, transcriptional evaluation by quantitative reverse transcription PCR of common genes identified in both transcriptomes and immunohistochemistry. The microarray data revealed 71 genes with differential expression in WAR, and the RNA-Seq data revealed 64 genes in GASH/Sal, showing common genes in both models. Analysis of transcripts showed that *Egr3* was overexpressed in WAR and GASH/Sal after audiogenic seizures. The *Npy, Rgs2, Ttr*, and *Abcb1a* genes presented the same transcriptional profile in the WAR, being overexpressed in the naïve and stimulated WAR in relation to their controls. *Npy* appeared overexpressed only in the naïve GASH/Sal compared to its control, while *Rgs2* and *Ttr* genes appeared overexpressed in naïve GASH/Sal and overexpressed after audiogenic seizure. No statistical difference was observed in the expression of *Abcb1a* in the GASH/Sal model. Compared to control animals, the immunohistochemical analysis of the inferior colliculus showed an increased immunoreactivity for NPY, RGS2, and TTR in both audiogenic models. Our data suggest that WAR and GASH/Sal strains have a difference in the timing of gene expression after seizure, in which GASH/Sal seems to respond more quickly. The transcriptional profile of the *Npy, Rgs2*, and *Ttr* genes under free-seizure conditions in both audiogenic models indicates an intrinsic expression already established in the strains. Our findings suggest that these genes may be causing small changes in different biological processes involved in seizure occurrence and response, and indirectly contributing to the susceptibility of the WAR and GASH/Sal models to audiogenic seizures.

## Introduction

Epilepsy is a neurological condition determined by enduring predisposition to generate seizures and by its neurobiological, cognitive, psychological, and social consequences (1, 2). Several experimental models (*in vivo, in vitro*, and *in silico*) are available for the study of this disorder, and their use has been essential for understanding the ictogenic and epileptogenic processes (3–5). Experimental animal models are indispensable for epilepsy research and can be developed through induction of seizures in wild-type animals or inbreeding genetically epilepsy-prone animals (6, 7). The genetically seizure-prone models are maintained using inbreeding protocols and have a genetic background with minimal or no variation. Thus, these models may provide important molecular and genetic clues to uncover the development of epilepsy and the occurrence of seizures. Among the genetic models of epilepsy, there are the audiogenic models whose animals are susceptible to seizures triggered by acoustic stimulation, named audiogenic seizures.

The Wistar Audiogenic Rat (WAR) and the Genetic Audiogenic Seizure Hamster from Salamanca (GASH/Sal) strains are the most recent audiogenic models in rodents (8, 9). Although they originate from different species, the WAR and GASH/Sal strains present common aspects. Both are genetic models sensitive to sound stimuli, the WAR strain developed by selective reproduction of Wistar animals and the GASH/Sal strain with DNA mutations arising in a colony of Syrian hamsters (8, 9). During acoustic stimulation (sound intensity 110–120 dB), WAR and GASH/Sal animals exhibit a latency period that is interrupted by a wild running and subsequent generalized tonic–clonic seizures that may progress to more severe motor manifestations, such as dorsoventral flexion of the neck and hyperextension of the forelimbs and hindlimbs (8, 9).

The inferior colliculus (IC) is the main structure involved in the development of seizures in the audiogenic models, considered the epileptogenic nucleus (8–11). Experiments in the WAR model demonstrated the importance of this structure in the initiation of the audiogenic seizures (12, 13), and the immunoreactivity of the neural activity marker, c-Fos, was observed in the IC of WAR and GASH/Sal animals following seizures (8, 14). In addition to the behavioral similarities, it is believed that other aspects are shared between the two audiogenic models. Neurochemical and molecular aspects have been investigated and suggest that GASH/Sal presents abnormalities in the GABAergic neurotransmission (15) as already proposed for the WAR model (16, 17). Furthermore, López-López et al. (18) revealed common molecular alterations, such as the overexpression of the *Egr1, Egr2*, and *Egr3* genes in the IC of both models.

Later on, the IC of stimulated GASH/Sal and Syrian hamsters were used in RNA sequencing (RNA-Seq) analyses (18). Based on these RNA-Seq data from GASH/Sal and the microarray data from WAR, other genes were identified as differentially regulated in both models.

In the current study, we evaluated the transcriptional profiles of these common genes by quantitative reverse transcription PCR (RT-qPCR) and the protein expression in the epileptogenic nucleus by immunohistochehstry. The molecular mechanisms underlying epileptogenesis are thought to be associated with altered expression of gene groups. We believe that identifying common expression patterns in these two audiogenic models of different species may help elucidate molecular mechanisms involved in the common phenotype: audiogenic seizures. Our results contribute to the molecular characterization of the mentioned models and understanding of biological processes involved in the predisposition or response to sound-triggered seizures. In addition, it would provide new molecular targets with potential implications for human epilepsy studies and drug development.

## Materials and Methods

### Animals

Fourteen WAR and 13 Wistar male rats 12 weeks old were obtained from the Animal's Facility of the University of São Paulo (USP, Brazil). Fourteen GASH/Sal and 13 male Syrian hamsters 10 weeks old were obtained from the Animal's Facility of the University of Salamanca (USAL, Spain) and from Janvier Labs (Le Genest-Saint-Isle, France), respectively. The age of the animals was previously defined considering the period of greatest phenotypic stability of both audiogenic models. In the WAR, the maximum audiogenic seizure susceptibility is since 10 weeks old (19). In the GASH/Sal, the maximum seizure susceptibility occurs between 2 and 4 months, and subsequently decays (20) to the point of losing seizure around 6 months old, probably due to a gradual hearing loss (8). All animals were maintained under normal conditions of lighting (12-h light/dark cycle) and temperature (22 ± 1°C) in an acoustically controlled environment, and with free access to water and food. The animals were subdivided into four groups: (1) the naïve control group (Wistar N and Syrian N; *n* = 7 per strain); (2) the naïve audiogenic group (WAR N and GASH/Sal N; *n* = 7 per strain) corresponding to seizure-prone animals that did not receive any acoustic stimulation or developed any seizures; (3) the stimulated control group (Wistar S and Syrian S; *n* = 6 per strain), composed of Wistar rats and Syrian hamsters subjected to acoustic stimulation; and (4) the stimulated audiogenic group (WAR S and GASH/Sal S; *n* = 7 per strain) corresponding to seizure-prone animals that were subjected to acoustic stimulation and presented a convulsive episode. The acoustic stimulation was individual and consists of a single high-intensity acoustic stimulus of 110 dB for a maximum of 60 s or until the onset of the tonic seizure in cases of the audiogenic animals. The stimulus is the sound of a ring bell recorded on an audiotape digitalized with a high-pass filter (>500 Hz) and reproduced by a computer coupled to amplifiers and tweeters in the upper side of the cage (120 dB) (21). All animals submitted to the stimulation were evaluated according to the severity index (SI) described by Garcia-Cairasco et al. (21, 22). The stimulated control group (Wistar S and Syrian S) was composed of animals that did not respond to sound. All rats and hamster controls presented SI = 0.00 (no seizures). The stimulated audiogenic seizures susceptible group (WAR S and GASH/Sal S) was composed of animals that presented generalized tonic–clonic seizures and clonic spasms. All of the WAR presented SI ≥ 0.85 (tonic seizures plus generalized clonic seizures). The euthanasia and tissue collection were defined according to the methodology employed. Euthanasia was 60 min after the acoustic stimulation for specific groups for qPCR transcript quantification and 90 min for immunohistochemical protein evaluation.

The tissue preparation of WAR animals and their control counterparts was carried out at the University of São Paulo following the same procedures used for the GASH/Sal at the University of Salamanca (see below), and all samples including brain tissues were appropriately shipped to the University of Salamanca to process all specimens in parallel. All procedures and experimental protocols were performed according to the guidelines of the European Community's Council Directive (2010/63/UE) and Brazilian legislation for the care and use of laboratory animals, and approved by the Bioethics Committee of the University of Salamanca (approval number 300) and the Ethics Committee on Animal Experimentation of the University of São Paulo.

### WAR Microarray

WAR microarray data were obtained during the study performed by López-López et al. (18). Briefly, RNA was extracted from the IC of seven WAR and three Wistar rats previously subjected to acoustic stimulation. After RNA purification and quantification, the RNA was amplified subsequently, using the WT Sense Target labeling and control reagents kit (Affymetrix Inc.), and then hybridized to rat microarrays (Gene 1.0 ST Array). Following scanning and image analysis, the microarray data were imported and the significance analysis of microarrays (SAM algorithm) was used to identify significant differential expression between the experimental groups. We selected the genes that vary in a range fold change greater than or equal to two (|FC| ≥ 2) among other genes and *p* < 0.05 (*p* ≤ 0.05). The functional annotation of the selected genes was performed using the Gene Ontology (GO) Consortium database. The microarray WAR data were deposited in the NCBI's Gene Expression Omnibus at GEO Series accession numbers GSE74150 (18).

### GASH/Sal RNA-Seq

The GASH/Sal RNA-Seq data were obtained from the study by López-López et al. (18). Briefly, RNA was extracted from the IC of four GASH/Sal and four Syrian hamsters that were subjected to acoustical stimulation as described above. After RNA purification and quantification, a pool of the RNA samples from IC of each group was generated. After validation and quantification of both libraries, the sequencing was performed in the Illumina Miseq platform (Illumina). Raw sequence was assessed for quality using FastQC and filtered by value ≥30 on the Phred scale (0–40). The filtered reads were mapped in reference genome of Syrian hamster, *Mesocricetus auratus* (MesAur 1.0), using STAR software, and the identification of differentially regulated genes was performed using EdgeR software. For this study, we selected the genes that vary in a range fold change greater than or equal to two (|FC| ≥ 2) and with a sum of reads greater than or equal to 100 (Σreads ≥ 100) between the sample sequenced in GASH/Sal and the control. Subsequently, the selected genes were submitted to functional annotation using the GO Consortium database. The data of RNA-Seq GASH/Sal were deposited in the NCBI BioProject at accession number 230618 (18).

### Quantitative Reverse Transcription PCR (RT-qPCR)

The primers were designed for species *Rattus norvegicus* and *M. auratus*. Gene sequences were obtained from the Ensembl Genome Browser database (http://www.ensembl.org/index.html) and the primers were designed aligned in different exons using the Primer3 software (http://bioinfo.ut.ee/primer3-0.4.0/primer3/) (Table 1). The quality of the designed primers was evaluated through NetPrimer software (http://www.premierbiosoft.com/netprimer/), and their specificity was tested by alignment analysis using the Primer-BLAST software (https://www.ncbi.nlm.nih.gov/tools/primer-blast/). The primers were synthesized by Thermo Fisher Custom Primers (Invitrogen–Thermo Fisher).

**Table 1:** Sequences of the primers designed for quantitative reverse transcription PCR analyses.

**Gene symbol**	**Ensembl number**	**Species**	**Forward (5^**′**^-3^**′**^)**	**Reverse (5^**′**^-3^**′**^)**	**Product size (bp)**
*Egr3*	ENSRNOG00000017828 ENSMAUG00000000747	*Rattus norvegicus* *Mesocricetus auratus*	CCACAAGCCCTTCCAGTGTC	GTGCGGATGTGAGTGGTGAG	75
*Npy*	ENSRNOG00000046449 ENSMAUG00000018791	*Rattus norvegicus* *Mesocricetus auratus*	ACCCTTCCATGTGGTGATGG	TGGACAGGCAGACTGGTTT	100
*Rgs2*	ENSRNOG00000003687 ENSMAUG00000020846	*Rattus norvegicus* *Mesocricetus auratus*	TGCTCTGGGCAGAAGCATTT	AAGTCTTCGCAAGCCAACCA	124
*Ttr*	ENSRNOG00000016275 ENSMAUG00000011770	*Rattus norvegicus* *Mesocricetus auratus*	GCCTCGCTGGACTGATATTTG	TCGGACAGCATCCAGGACTT	95
*Abcb1a*	ENSRNOG00000008012 ENSMAUG00000012772	*Rattus norvegicus* *Mesocricetus auratus*	GGAAATCATTGGGGTGGTGA	GGCATTGGCTTCCTTGACAG	127
Actb^*^	ENSRNOG00000034254 ENSMAUG00000008763	*Rattus norvegicus* *Mesocricetus auratus*	AGCCATGTACGTAGCCATCC	ACCCTCATAGATGGGCACAG	115

* *Housekeeping gene is selected as the reference gene*.

A total of 48 animals (6 animals from each group and each strain) were used for mRNA analysis of the IC. All animals were euthanized 60 min after the stimulus of the specific groups. RNA from IC samples was extracted according to the protocol of TRIzol™ Reagent (#15596026, Invitrogen). The total RNA was quantified using the Spectrophotometer NanoDrop 2000 (Thermo Fisher Scientific), and the samples used presented 260/230 nm and 260/280 nm ≥ 1.8 ratios. The complementary DNA (cDNA) synthesis was performed using the First Strand cDNA Synthesis Kit (#K1622, Promega).

The relative quantification of the transcripts was performed on ABI Prism 7000 (Applied Biosystems) using the SYBR Green Master Mix (#4309155, Applied Biosystems). The list of primers used is provided in Table 1. The housekeeping *Actb* gene (β-actin) was selected as the reference gene. Initially, two candidate genes [β-actin (*Actb*) and glyceraldehyde 3-phosphate dehydrogenase (*Gapdh*)] were selected according to the expression levels detected in the WAR and GASH/Sal microarray (18). The expression of these genes was also verified by RT-qPCR, and the NormFinder software was used to calculate the intra- and intergroup gene expression variations. Results indicated *Actb* as the most stable gene for normalization of RT-qPCR data in the IC.

Initially, a serial dilution curve was made to verify the efficiency of the primers of the target and reference genes. The plates were assembled by species and the reactions were evaluated in duplicate containing the following: 10 μl of SYBR, 2 μl of cDNA (10 ng), 0.4 μl of each primer (10 μM), and 7.2 μl of MiliQ water free of DNase and RNase completing the volume of 20 μl. The cycling conditions were according to the protocol of the intercalating agent used. The relative gene expression value for each transcript was calculated according to the formula 2^−(ΔCt “condition 1”−ΔCt “condition 2”)^, where “condition 1” corresponds to the experimental sample, “condition 2” corresponds to the sample from the control animal, and ΔCt of each “condition” is Ct_“experimental gene”_ − Ct_“endogenous gene”_ (23). Quantification data were analyzed for normality distribution using the D'Agostino-Pearson and Shapiro-Wilk tests. The relative mRNA of the groups was evaluated using unpaired *t*-test. The analyses were performed using GraphPad Prism 7 and outlier analyses were performed by Grubbs test. *p* ≤ 0.05 was considered as statistically significant.

### Immunohistochemistry

A total of six animals corresponding to the control naïve group (one animal per strain) as well as audiogenic animals at naïve (one animal per strain) and sound-stimulated (one animal per strain) conditions were used for immunohistochemical analysis of the proteins encoded by the *Npy, Rgs2*, and *Ttr* genes. The animals were euthanized with phenobarbital (60 mg/kg) and transcardially perfused with an aldehyde-based fixative following the standard laboratory protocols (24). To verify gene expression related to audiogenic seizures, the perfusion protocol was carried out 90 min after the sound stimulation of the specific group. The brains from all experimental groups were processed in parallel using the same procedures and reagents for light microscopy study. Briefly, the brains were removed from the skull without the meninges, cryoprotected by immersion in 30% sucrose and sectioned in coronal sections of 40 μm thickness using a freezing sliding microtome MICROM HM 400R (Leica Biosystems). The free-floating sections were washed and treated in blocking solution to subsequently incubate in primary and secondary antibody antisera (Table 2) following the indirect detection method (24). Then, the sections were washed and treated for the chromogenic detection in the ABC/DAB system without nickel intensification (#PK-4000 and #SK-4100, Vector Laboratories). For each brain, the sections were mounted on slides, dehydrated in ethanol, and coverslipped with Entellan Neu (#107961, Merck). Negative controls were not treated with primary antibodies, and this resulted in no immunolabeling. Histological sections containing the IC were examined using a microscope DMBL (Leica Biosystems) equipped with a digital camera DP50 (Olympus). Low- and high-magnification photomicrographs were captured with 10 × and 40 × objective lens, respectively. All images were adjusted with minor modifications for brightness and contrast using Adobe Photoshop (version 9.0), and the final composition of the figures was achieved with Canvas 14 software.

**Table 2:** Antibodies and dilutions used for the immunohistochemical approaches.

**Antigen**	**Primary antibody**	**Reference**	**Dilution**	**Secondary antibody**	**Reference**	**Dilution**
NPY	Sheep anti-Neuropeptide Y	ab6173 Abcam	1/500	Biotinylated rabbit Anti-Sheep	BA-6000 Vector	1/200
RGS2	Rabbit anti-RGS2	ab36561 Abcam	1/300	Biotinylated goat Anti-Rabbit	BA-1000 Vector	1/200
TTR	Rabbit anti-Prealbumin	ab9015 Abcam	1/1,000	Biotinylated goat Anti-Rabbit	BA-1000 Vector	1/200

## Results

### Differentially Expressed Genes and Functional Annotation

**Figure 1:**
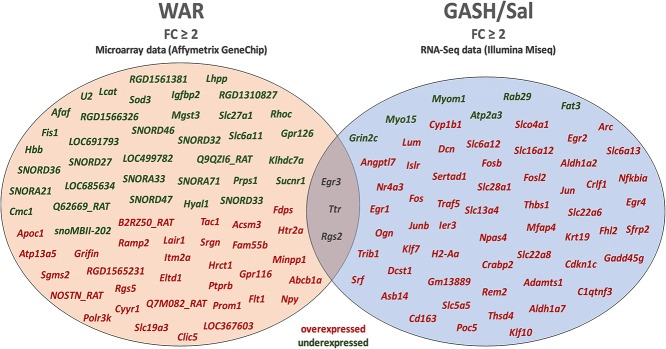
Diagram with the genes identified as differentially regulated in the inferior colliculus (IC) of the WAR and GASH/Sal models using the selection criterion of fold change (|FC| ≥ 2). cDNA microarray analysis and RNA-Seq identified a total of 71 and 64 differentially expressed genes in the WAR and GASH/Sal, respectively. A set of three differentially expressed genes (*Egr3, Ttr*, and *Rgs2*) overlapped between the microarray and RNA-Seq approaches. Genes highlighted in red: overexpressed genes; Genes highlighted in green: underexpressed genes; Genes highlighted in gray: common differentially expressed genes.

**Figure 2:**
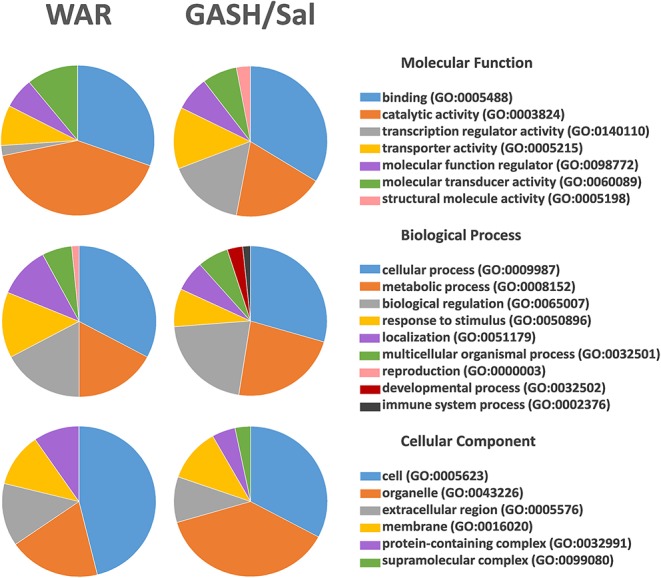
Functional annotation of the genes identified as differentially regulated in WAR and GASH/Sal models. The functional annotation was performed, independently, with the 71 differentially expressed genes in the WAR model and 64 differentially expressed genes in the GASH/Sal model. The annotation presents categories of molecular functions, biological processes, and cellular components that are represented by different colors on the pie charts.

**Figure 3:**
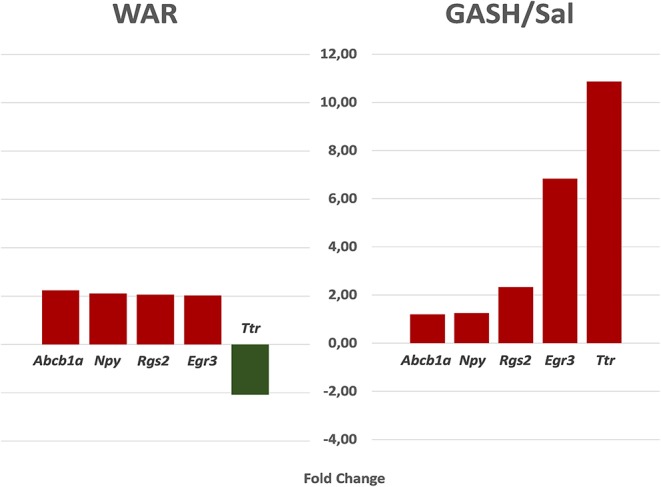
The fold change (FC) of the genes evaluated by RT-qPCR according to transcriptome data. *Egr3, Rgs2*, and *Ttr* genes are commonly differentially regulated between WAR and GASH/Sal models, and *Npy* and *Abcb1a* are not filtered in the GASH/Sal data by the FC criterion, but included in this evaluation due to preliminary results. In the WAR model: *Abcb1a* (FC = 2.23); *Npy* (FC = 2.10); *Rgs2* (FC = 2.06); *Egr3* (FC = 2.07); *Ttr* (FC = −2.08). In the GASH/Sal model: *Abcb1a* (FC = 1.19); *Npy* (FC = 1.25); *Rgs2* (FC = 2.33); *Egr3* (FC = 6.83); *Ttr* (FC = 10.86).

The stimulated WAR and GASH/Sal transcriptomes allowed us to identify common differentially regulated genes between these audiogenic models. The microarray data from WAR revealed 71 genes with differential expression considering FC values ≥2 and the RNA-Seq data from GASH/Sal revealed 64 genes considering the same FC. The genes Early Growth Response 3 (*Egr3*), Regulator of G-protein signaling 2 (*Rgs2*), and Transthyretin (*Ttr*) were differentially expressed in both models (Figure 1). The *Egr3* gene encodes a transcription factor that is induced by several stress factors. The *Rgs2* gene encodes a protein that modulates G protein-coupled receptor signaling cascades. The *Ttr* gene encodes a carrier protein that transports the thyroxin and retinol binding protein complex, and is involved in neuropathies. The functional annotation of the genes selected from each model showed common categories of molecular functions, biological processes, and cellular components. Among the molecular functions, the categories “binding” and “catalytic activity” stood out in both models, presenting the highest number of genes with differential expression. Furthermore, it is noteworthy that the category “transcription regulator activity” was found the third most represented in GASH/Sal, showing 11 differentially expressed genes, Early Growth Response 1, 2, 3, and 4 (*Egr1*; *Egr2*; *Egr3*; *Egr4*), SERTA domain containing 1 (*Sertad1*), Neuronal PAS domain protein 4 (*Npas4*), TNF receptor associated factor 5 (*Traf5*), Kruppel-like factor 7 and 10 (*Klf7; Klf10*), and AP-1 transcription factor subunit (*Jun; Junb*), whereas in WAR, the same category was represented only by the *Egr3* gene. In the classification of biological processes, the categories “cellular process” and “metabolic process” were the most represented in both models followed by the categories “biological regulation” and “response to stimulus” (Figure 2). The common differentially expressed genes between WAR and GASH/Sal were mainly in the categories “metabolic process,” “catalytic activity,” and “transcriptional regulator activity.” The *Egr3, Rgs2*, and *Ttr* genes were identified as overexpressed in RNA-Seq data of the GASH/Sal strain, and the *Egr3* and *Rgs2* genes were identified as overexpressed in microarray data from WAR, while the *Ttr* gene was shown underexpressed. To validate these data, we quantified the transcript levels of the common genes between the models. Neuropeptide Y (*Npy*) and ATP binding cassette subfamily B member 1A (*Abcb1a*) genes were also assessed, both overexpressed in WAR and GASH/Sal (Figure 3). *Npy* encodes the neuropeptide Y that acts as a neurotransmitter and is involved in physiological and homeostatic processes. *Abcb1a* encodes a transmembrane glycoprotein related to multidrug resistance. *Npy* and *Abcb1a* were not filtered in the GASH/Sal data by the FC criterion. However, we included them in this study due to preliminary results of these genes in WAR (unpublished data) and potential in the epileptic phenotype and treatment, respectively.

### Quantitative Reverse Transcription PCR (RT-qPCR)

**Figure 4:**
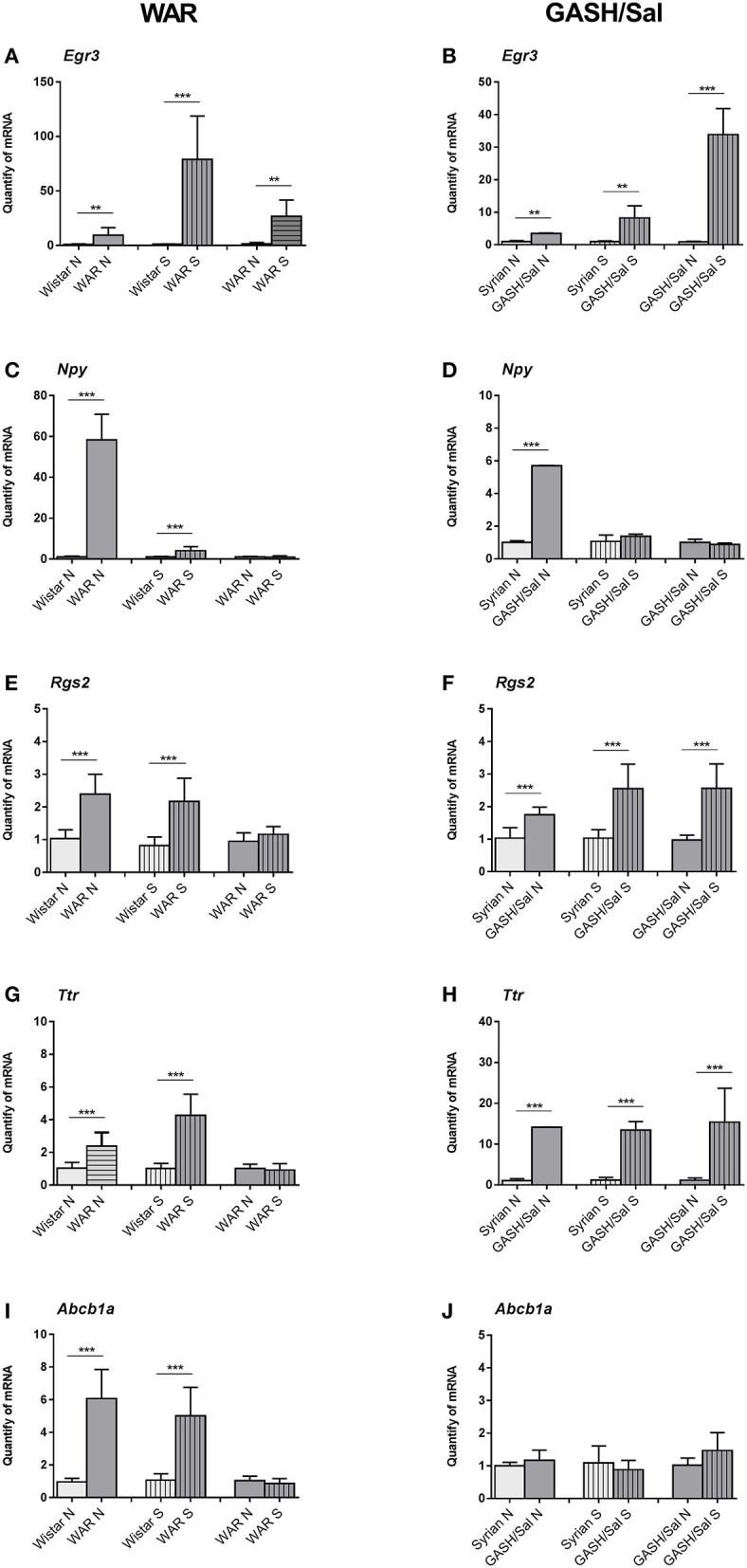
Relative quantities of transcripts in the inferior colliculus (IC) of the WAR and GASH/Sal models. (Left) WAR Graphics; (Right) GASH/Sal Graphics. In the graphics, *X*-axis: Relative quantities of mRNA in arbitrary units, *Y*-axis: Experimental groups: naïve control group (Wistar N; Syrian N); stimulated control group (Wistar S; Syrian S); naïve audiogenic group (WAR N; GASH N); stimulated audiogenic group that presented seizures (WAR S; GASH S). **(A,B)** *Egr3* gene is overexpressed in naïve WAR and GASH/Sal and after seizure. **(C,D)** *Npy* gene is overexpressed in both naïve audiogenic groups compared to their respective controls. No statistical difference is observed in the *Npy* expression between the naïve and stimulated audiogenic groups. **(E,F)** *Rgs2* gene is overexpressed in WAR and GASH/Sal compared to their respective controls, and overexpressed after seizure just in the GASH/Sal model. **(G,H)** *Ttr* gene presents overexpression in the naïve and stimulated audiogenic groups in relation to their respective controls, and overexpressed after seizure just in the GASH/Sal model. **(I,J)** *Abcb1a* gene is overexpressed in WAR model and no statistical difference was observed in the expression of *Abcb1a* in the GASH/Sal model. Bars represent mean ± SEM. Statistical analyses: Unpaired *t*-test. **p ≤ 0.01, or ***p ≤ 0.001.

The evaluation of genes by RT-qPCR was performed with naïve groups (Wistar N and WAR N; Syrian N and GASH/Sal N) and groups of animals subjected to acoustic stimulation (Wistar S and WAR S; Syrian S and GASH/Sal S). Comparisons between naïve and sound-stimulated groups allowed us to validate the transcriptional profile observed in the transcriptome data according to the comparisons of Wistar S vs. WAR S and Syrian S vs. GASH/Sal S. Also, comparisons of mRNA gene expression analysis between Wistar N vs. WAR N and Syrian N vs. GASH/Sal N verified whether differential regulation is inherent to each model. By comparing between WAR N vs. WAR S and GASH/Sal N vs. GASH/Sal S, the RT-qPCR analysis determined whether the occurrence of seizures modulates gene transcription. The *Egr3* gene presented the same transcriptional profile in the WAR and GASH/Sal models, showing overexpression in the naïve and stimulated audiogenic groups in relation to their respective controls and overexpressed after acoustic stimulation followed by seizures (Figures 4A,B). The *Npy* gene was overexpressed in the WAR N and GASH/Sal N groups, when compared to their respective controls. No statistical difference was observed in the *Npy* expression between the naïve audiogenic groups (WAR N; GASH/Sal N) and stimulated audiogenic groups (WAR S; GASH/Sal S) (Figures 4C,D). The *Rgs2* gene in the WAR model presented a transcriptional profile similar to the *Npy* gene, being overexpressed in the WAR independently of the acoustic stimulus. This profile was observed by the overexpression of *Rgs2* in the WAR N and WAR S groups in comparison to their controls and by the absence of statistical difference between WAR N and WAR S (Figure 4E). *Rgs2* showed overexpression in GASH/Sal N and GASH/Sal S in relation to their controls, and overexpression in GASH/Sal S compared to GASH/Sal N (Figure 4F). The *Ttr* gene was overexpressed in the naïve and stimulated audiogenic groups (WAR N, WAR S, GASH/Sal N, and GASH/Sal S) in relation to their respective controls. In WAR, there was no statistical difference in the *Ttr* expression between WAR N and WAR S groups while it was overexpressed in the GASH S group in comparison to the GASH N group (Figures 4G,H). The *Abcb1a* gene was overexpressed in the WAR N and WAR S compared to controls (Figure 4I) while no statistical difference was observed in the expression of *Abcb1a* in the GASH/Sal model (Figure 4J).

### NPY, RGS2, and TTR-Immunoreactivity in the IC

**Figure 5:**
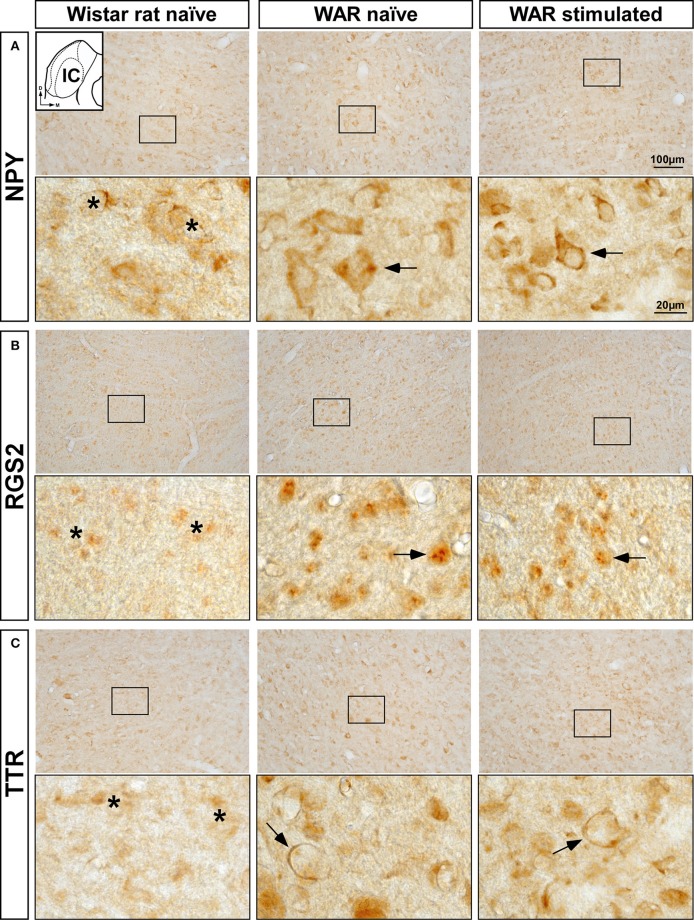
Immunolabeling of NPY, RGS2, and TTR proteins in the inferior colliculus (IC) of the control Wistar rat and the WAR model at naïve and sound-stimulated conditions. The inset in **(A)** depicts a representative schematic section of the IC (8.28 mm caudal from bregma), in which the immunoreactivity was analyzed. For each protein, upper panels show lower-magnification photomicrographs of the IC and lower panels show higher magnifications corresponding to the frame in the upper panels. **(A)** Numerous and strong NPY-immunopositive perikarya (arrows) are shown in WAR animals at naïve and sound-stimulated conditions, whereas naïve Wistar rats exhibit sparse and weak NPY-immunolabeling (asterisks). **(B)** Strong nuclear dot-like immunolabeling for RGS2 protein (arrows) is found in WAR animals at naïve and sound-stimulated conditions. Notice the discrete or absence of RGS2-immunolabeling (asterisks) in the control animal. **(C)** An increase in immunolabeling of TTR (arrows) is shown in WAR animals at naïve and sound-stimulated conditions when compared to naïve Wistar rats (asterisks). Notice the distribution of TTR-immunolabeling outlining the cell bodies of unlabeled IC neurons (arrows). Scale bars for upper panels and lower panels are 100 and 20 μm, respectively.

**Figure 6:**
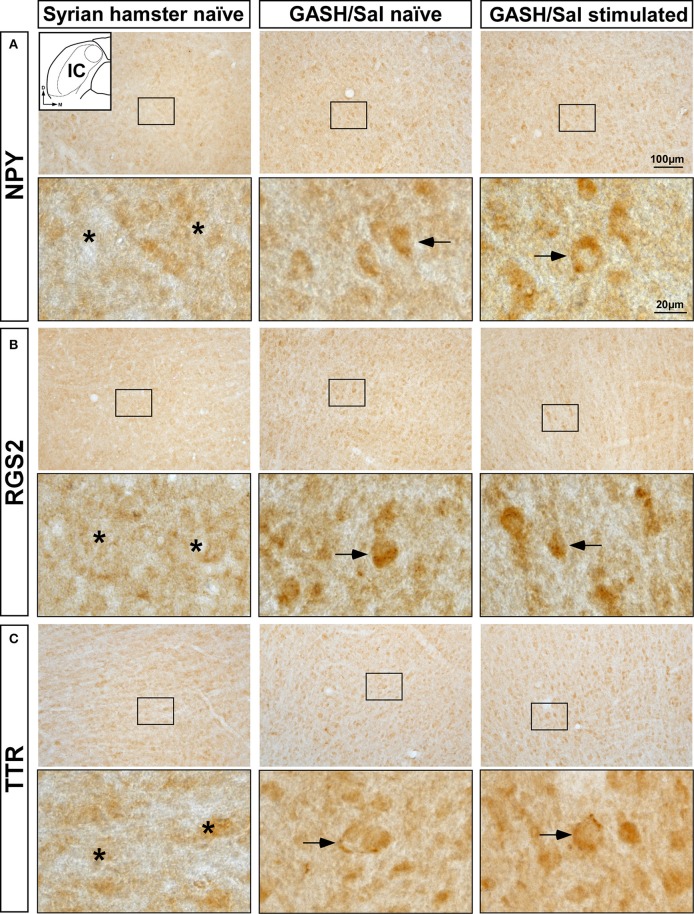
Immunolabeling of NPY, RGS2, and TTR proteins in the inferior colliculus (IC) of the control Syrian hamster and the GASH/Sal model at naïve and sound-stimulated conditions. The inset in **(A)** depicts a representative schematic section of the IC (6.00 mm caudal from bregma), in which the immunoreactivity was analyzed. For each protein, upper panels show lower-magnification photomicrographs of the IC and lower panels show higher magnifications corresponding to the frame in the upper panels. **(A)** Weak NPY-immunopositive perikarya (arrows) are shown in GASH/Sal animals at naïve and sound-stimulated conditions, whereas naïve control Syrian hamster exhibit absence of NPY-immunolabeling (asterisks). **(B)** Moderate nuclear immunolabeling for RGS2 protein (arrows) is found in GASH/Sal animals at naïve and sound-stimulated conditions. Notice the absence of RGS2-immunolabeling (asterisks) in the control animal. **(C)** An increase in immunolabeling of TTR (arrows) is shown in GASH/Sal animals at naïve and sound-stimulated conditions when compared to naïve Syrian hamsters (asterisks). Notice the distribution of TTR-immunolabeling outlining the cell bodies of unlabeled IC neurons (arrows). Scale bars for upper panels and lower panels are 100 and 20 μm, respectively.

The increased mRNA expression levels of *Npy, Rgs2*, and *Ttr* genes in the WAR and GASH/Sal models led us to analyze the immunolabeling of the corresponding encoded proteins in the IC. Visual qualitative comparisons of the NPY, RGS2, and TTR-immunoreactive patterns were carried out between control naïve animals and both audiogenic seizure strains at naïve and sound-stimulated conditions. The most noticeable difference between the audiogenic seizure brains and their control counterparts was an increased immunoreactivity for NPY, RGS2, and TTR in the IC of WAR and GASH/Sal animals (Figures 5, 6). Strong NPY-immunoreactivity was found in the perikarya of numerous IC neurons in WAR animals, whereas the Wistar rats showed weak immunolabeling. These NPY-immunoreactive cell bodies were particularly dense in WAR animals that received sound stimulation (Figure 5A). Very few RGS2 nuclear immunolabeling was found in Wistar control rats; conversely, the IC of WAR animals at naïve and sound-stimulated conditions showed strong nuclear dot-like immunolabeling (Figure 5B). Immunolabeling pattern of TTR was found outlining the cell bodies of IC neurons, suggesting that TTR protein distributed in the cell membrane or the outer surface of the plasma membrane (Figure 5C). An increase in cytoplasmic immunolabeling of TTR was seen in WAR animals at naïve and sound-stimulated conditions when compared to naïve Wistar rats that exhibited absence or discrete immunolabeling (Figure 5C). The pattern of distribution of NPY, RGS2, and TTR in the IC of GASH/Sal animals was very similar to that observed in WAR animals, but with the particularity that the intensity of immunolabeling was greater in WAR animals (see Figures 5, 6 for comparisons). In hamsters, NYP-immunolabeled perikarya were seen in GASH/Sal animals, particularly those animals at sound-stimulation conditions, while Syrian controls exhibited absence of immunolabeling (Figure 6A). As shown in WAR animals, the RGS2 and TTR-immunolabeling were stronger in GASH/Sal animals than that observed in control Syrian hamsters (Figures 6B,C). Together the immunohistochemistry results correlated with RT-qPCR analysis, indicating an overexpression of NPY, RGS2, and TTR proteins in the IC of WAR and GASH/Sal animals.

## Discussion

Transcriptional profiling of an experimental animal model is a strategy that aims to identify differentially regulated genes in a given space and time. This approach helps to identify altered molecular processes and investigate causal or predisposing factors associated with seizure pathology. In the present study, we evaluated the transcriptional profile of the epileptogenic nucleus in the WAR and GASH/Sal models after sound-induced seizures. Since WAR and GASH/Sal are audiogenic seizure models in different species but with similar epileptic phenotype, the identification of common differentially regulated genes allows us to infer the possible molecular alterations underlying the audiogenic phenotype.

López-López et al. (18) carried out the first comparative study between the WAR and GASH/Sal transcriptional profiles using the microarray methodology. In this study, the reactions were performed with mouse GeneChip due to the lack of GeneChip for Golden hamster, which resulted in a reduced number of identified genes. Among the 15 differentially transcribed genes detected in the GASH/Sal model considering a |FC| ≥ 1.5, only the *Egr3* gene was identified in common with the WAR model. Latterly, a new transcriptome of the epileptogenic nucleus in the GASH/Sal was established using RNA-Seq as a more robust and accurate transcriptomic approach (18). With the availability of these data, we currently performed a new comparative study between the WAR and GASH/Sal transcriptomes.

The microarray data from WAR were analyzed and the differentially expressed genes were selected based on fold change (|FC| ≥ 2) with a *p-*value (*p* ≤ 0.05). In the RNA-Seq data from GASH/Sal, the differentially expressed genes were selected based on the same fold change (|FC| ≥ 2) and sum of reads (Σreads ≥ 100). The selection by *p-*value was not possible because the sequencing was performed with two samples composed of pooled RNAs from different animals of each group. These screenings revealed 71 genes in the WAR and 64 genes in the GASH/Sal model with differential expression.

The functional annotation of the differentially expressed genes in each model showed that part of these genes is included in important categories that may indicate metabolism dysfunctions, such as “catalytic activity,” “metabolic processes,” and “biological regulation.” In another study, a similar ontological profile was observed in the WAR model, in which metabolic alterations were also suggested (25). In the clustering of molecular functions, we observed that the GASH/Sal model has a large representation of genes in the “transcription regulator activity” category that was not observed in the transcriptome of the WAR model. This difference between both audiogenic models suggests that the time for gene expression after sound-induced seizure might be faster in GASH/Sal than in WAR animals, even though the IC collection was performed at the same interval time for the two audiogenic strains (60 min).

Comparison between WAR and GASH/Sal transcriptomes revealed the *Egr3, Rgs2*, and *Ttr* genes as differentially expressed in both audiogenic models. These were evaluated by RT-qPCR together with the *Npy* and *Abcb1a* genes. These two genes were included in the present study using unpublished data from previous gene expression analysis. The quantification of target gene transcripts confirmed the differential expression of *Egr3, Npy, Rgs2*, and *Ttr* in both models, while *Abcb1a* showed differential expression in the WAR model, but not in the GASH/Sal. The *Egr3* gene showed overexpressed in the audiogenic animals at naïve and sound-stimulated conditions when compared with their respective controls, and overexpressed after sound-induced seizures. The *Egr3* gene encodes a transcription factor that is constitutively expressed in diverse telencephalic regions including the neocortex, striatum, piriform cortex, amygdala, septum, and hippocampus, according to results of *in situ* hybridization in the rat forebrain (26). *Egr3* gene expression is induced rapidly and transiently in the cortex and hippocampus after electroconvulsive seizures (26) and increased in the IC, hippocampus, and ventral cochlear nucleus after audiogenic seizures (18). *Egr3*, together with other genes of the same family of “early growth response,” has already been assessed in WAR and GASH/Sal, showing an overexpression in the IC of both animal models (18).

The transcriptional profile of *Npy* revealed that its overexpression in the IC is intrinsic to each model and independent of seizures, since we did not observe expression differences between naïve and stimulated epileptic animals. Brain sections showed a higher expression of NPY in the WAR and GASH/Sal animals compared to their respective controls, which corroborates the transcriptional data of the two audiogenic models. NPY acts as a neurotransmitter through its five G protein-coupled receptors (Y1, Y2, Y4, Y5, and Y6) and is constitutively expressed in multiple neuronal populations across fore, mid, and hindbrain (27, 28). One of the structures with significant NPY expression is the IC, principally in its external layer, according to immunohistochemistry and in situ hybridization results in the rat (27, 28). Moderate NPY expression was observed in the cuneiform nucleus and the periaqueductal gray, both located close and ventrally to the IC, while the rest of the midbrain structures, such as the superior colliculus, substantia nigra, ventral tegmental area, and dorsal raphe, are negative for NPY expression (29, 30).

The majority of studies describe the NPY acting as an anticonvulsant and report that seizures increase NPY expression in cortical and limbic structures, such as frontal cortex, hippocampus, and amygdala (31–36). This overexpression is generally considered an adaptive mechanism designed to contain the hyperexcitability underlying epileptiform activity and protect the brain from further insults (32, 33). The presynaptic receptor Y2 is thought to be the receptor at which NPY exerts its anticonvulsant action, since its activation has inhibitory effect on glutamate release (31, 33, 37). In contrast, the proconvulsant action of NPY has also been reported considering the involvement of the post-synaptic receptor Y1 (37–39). The distribution of these receptors seems to be regional. While Y2 receptors are more abundant in the hippocampus than in the neocortex, the opposite is observed for the Y1 receptor, which is more abundant in the neocortex than in the hippocampus (40–42). Although there is no detailed description of the distribution of NPY receptors in the IC, our immunohistochemical results showed that both audiogenic strains overexpressed the NPY protein in the cell bodies of IC neurons. This suggests that neurotransmission mediated by NPY is altered in the IC and possibly contributing to the seizure threshold in these audiogenic models.

In RT-qPCR evaluation, the *Rgs2* and *Ttr* genes presented a transcriptional profile similar to the *Npy* gene in the WAR model, being overexpressed in the WAR regardless of the stimulus and the occurrence of seizures. However, the microarray data indicated that the *Ttr* gene was underexpressed in sound-stimulated WAR animals compared to the control Wistar rats with the same stimulation. Differences between RT-qPCR and microarray experiments occur for several reasons, including the fact that different probes are used for the microarray and RT-qPCR experiments (which can capture differential expression in splice variants), differences in the methods for normalization of expression data and possible false-positive expression changes (43), and the lower accuracy of the microarray data. Therefore, it should be considered the result achieved with RT-qPCR, which is a more robust technique. In the GASH/Sal model, the *Rgs2* and *Ttr* genes were also overexpressed in relation to the control hamsters and showed an increment of the expression after the occurrence of seizures.

*Rgs2* and *Ttr* are involved in different molecular processes. The *Rgs2* gene encodes a protein that modulates G protein-coupled receptor signaling cascades (GPCRs), the so-called G protein signaling regulator 2 (RGS2). Upon activation, GPCRs catalyze the exchange of GDP for GTP into a coupled heterotrimeric G protein, promoting dissociation into free subunits (G-α and the G-βγ dimer), which allows to regulate downstream effector activities, such as adenylate cyclase regulation, calcium channels, potassium channels, phospholipase C, and mitogen-activated protein kinase pathway (44, 45). In general, RGSs proteins accelerate the intrinsic GTPase activity of the G-α subunit, causing GTP hydrolysis that in turn resulted in a reassociation of subunits (G protein in its inactive state), which abolishes or decreases the signal transduction induced by GPCRs (44, 45). *Rgs2* is highly expressed in the brain, and its overexpression is generally considered a rapid and transient response after changes in homeostasis (46–48). Increased levels of *Rgs2* transcripts were also observed in the prefrontal cortex and hippocampus of rats following an acute electroconvulsive stimulation and were normalized 24 h later (49). Such fluctuations in mRNA expression levels of *Rgs2* were suggested as an adaptation of neurons in an attempt to reduce cell depolarization and the probability of new epileptiform events (49), mainly because RGS2 indirectly modulates calcium channels (50, 51). Christensen et al. (52) also reported an increased expression of *Rgs2* in the hippocampus of kindled rats after electroconvulsive stimulation, showing that transcript levels of this gene were reduced after Levetiracetam treatment. These data support a possible involvement of *Rgs2* in the epileptogenic process.

The *Ttr* gene encodes a protein called transthyretin that is mainly synthesized by the liver and choroid plexus and is secreted in the blood and cerebrospinal fluid, respectively (53). TTR expression in the brain is mainly localized in the ependymal cells of the choroid plexus, appearing additionally in the meninges as well as in neurons under stress conditions or pathological conditions (54–58). The transthyretin is organized as functional homotetramers (TTRs) that are capable of transporting thyroxin hormone and retinol binding protein complex (59). Mutations in this gene may impair the association of these tetramers and promote the formation and deposition of amyloid fibrils in leptomeninges, causing leptomeningeal amyloidosis, a neuropathy characterized by slowly progressive dementia, seizures, ataxia, and subarachnoid hemorrhage (60–63). TTR is also related to other neuropathies. Decreased levels of this protein were observed in patients with amyotrophic lateral sclerosis, in which it acts as a biomarker (64), and in the cerebrospinal fluid of patients with Alzheimer's disease (65–67). Several studies have reported the neuroprotective role of TTR in patients with Alzheimer's (68–70) as well as in animal models of oligemia (71), in the animal model of Alzheimer's in which TTR expression assists in protection against induced neuronal death by beta amyloid (72), and in the animal model of induced seizures by cobalt (73).

Consistently with this neuroprotective effect, our results showed an overexpression of *Rgs2* and *Ttr* genes in GASH/Sal animals that suffered from sound-induced seizures in comparison with those at naïve conditions. This overexpression of *Rgs2* and *Ttr* genes after seizures was not found in WAR animals and, as would be expected, was not detected at the protein level in the IC sections of both models. Such differences might be explained because both audiogenic models seem to differ in the response time for gene expression after the sound-induced seizures. Therefore, a longer time to assess gene expression of *Rgs2* and *Ttr* might be necessary to found similar results between WAR and GASH/Sal animals at naïve and sound-stimulated conditions. As observed in the *Npy* gene, the most noticeable similarity between both audiogenic strains was the marked overexpression of *Rgs2* and *Ttr* genes when compared with their respective naïve controls. Consistent with this gene expression result, we found that RGS2 and TTR-immunolabeling was more intense in the IC of WAR and GASH/Sal animals than in their naïve controls. As described above, RGS proteins regulate GPCR-induced signaling and thus indirectly modulate various processes including activation of calcium and potassium channels. Also, a recent study reported that TTR regulates expression and function of the extra-synaptic GABAA receptors (GABAA-α*βδ*), which play an important role of tonic inhibition to regulate neuronal excitability in the brain (56). Our results showed a strong TTR-immunolabeling distributed in the cell membrane and surface of IC neurons of WAR and GASH/Sal animals. The same distribution pattern of TTR-immunolabeling was found in cerebellar granule neurons, in which TTR protein colocalized with GABAA receptors to modulate specific subunits (53). In this context, the increasing mRNA expression levels and immunoreactivity of RGS2 and TTR in the epileptogenic nucleus of WAR and GASH/Sal models may cause calcium and potassium dysfunctions as well as deficits in GABAA receptor-mediated neurotransmission, contributing to seizure susceptibility and development.

*Abcb1a* showed overexpression only in WAR animals when compared to controls. No differential expression was observed between GASH/Sal animals and their controls. *Abcb1a* is part of the MDR family (*Multiple Drug Resistance)* related to multidrug resistance. This gene encodes the P glycoprotein (PGP), a highly expressed transmembrane protein in the endothelial cells of the blood–brain barrier and that have functions related to excretion and/or protection of tissues against toxins and xenobiotics (74–76). Studies reported overexpression of PGP in different brain regions as a transient response of epileptic seizures, which occurs as an indirect consequence due to excess of glutamate released by increased neuronal activity (76–80). In the audiogenic genetically epilepsy-prone rat (GEPR), increased expression of *Abcb1a* in cortex and midbrain was observed 1 day after seizure (81). This high expression was not observed in the IC of WAR animals considering 60 min after the insult. Thus, we believe that it would take more time to observe the expression of this gene after the seizure. However, *Abcb1a* overexpression in naïve and stimulated WAR compared to controls indicates the inherent strain's characteristics. Tishler et al. (82) described increased levels of *Abcb1* gene transcripts in the brain of drug-resistant epilepsy patients and other authors have suggested that overexpression of PGP causes less penetration of antiepileptic drugs into the brain, which would reduce their effects (77, 82–84). In this context, the transcriptional profile of *Abcb1a* in WAR opens the possibility of using this model in future studies involving antiepileptic drugs and drug resistance.

The comparison between WAR and GASH/Sal transcriptomes allowed us to identify common differentially expressed genes and similar transcriptional profiles. The *Npy, Rgs2*, and *Ttr* genes were overexpressed in both audiogenic models compared to their basal state controls. This profile and protein immunostaining suggest that overexpression of these genes is intrinsic to each strain and probably this pattern was established during the decades of generations of reproductive selection of each model. The *Rgs2* and *Ttr* genes showed differential expression in the GASH/Sal model after the occurrence of seizure, which was not observed in WAR. In addition, the discrepant representation of genes in the “transcription regulator activity” category suggests that WAR and GASH/Sal do not have exactly the same response time to the insult. In summary, we consider that these differentially expressed genes in both audiogenic models cause changes in different biological processes involved in the occurrence and response to seizures and thus indirectly contributing to the susceptibility of WAR and GASH/Sal models to seizures.

## Data Availability Statement

The data obtained and discussed in this publication have been deposited in the NCBI's Gene Expression Omnibus at GEO Series accession numbers GSE74150 (http://www.ncbi.nlm.nih.gov/geo/query/acc.cgi?acc=GSE74150) for the WAR arrays and GSE74043 (http://www.ncbi.nlm.nih.gov/geo/query/acc.cgi?acc=GSE74043) for the GASH:Sal arrays.

## Ethics Statement

The animal study was reviewed and approved by Ethics Committee on Animal Experimentation of the University of São Paulo and Bioethics Committee of the University of Salamanca.

## Author Contributions

SD conducted all the experiments and wrote the paper. NG-C provided rats and dissected tissues from the breeding WAR colony in Brazil and corrected the manuscript. MH-T assisted in the RT-qPCR experiments. RG-N assisted in the immunostaining experiments and revised manuscript. FM assisted in the anatomical distribution of the proteins encoded by the studied genes. DL designed and supervised all the study and corrected the manuscript.

### Conflict of Interest

The authors declare that the research was conducted in the absence of any commercial or financial relationships that could be construed as a potential conflict of interest.

## References

[1] Fisher RS Acevedo C Arzimanoglou A Bogacz A Cross JH Elger CE. ILAE Official report: a practical clinical definition of epilepsy. Epilepsia. (2014) 55:475–82. 10.1111/epi.12550 24730690

[2] Fisher RS. Redefining epilepsy. Curr Opin Neurol. (2015) 28:130–5. 10.1097/WCO.0000000000000174 25734953

[3] Kandratavicius L Alves Balista P Lopes-Aguiar C Ruggiero RN Umeoka EH Garcia-Cairasco N. Animal models of epilepsy: use and limitations. Neuropsychiatr Dis Treat. (2014) 10:1693–705. 10.2147/NDT.S50371 25228809 PMC4164293

[4] Raimondo J V Heinemann U de Curtis M Goodkin HP Dulla CG Janigro D. Methodological standards for *in vitro* models of epilepsy and epileptic seizures. a TASK1-WG4 report of the AES/ILAE translational task force of the ILAE. Epilepsia. (2017) 58:40–52. 10.1111/epi.13901 29105075 PMC5679463

[5] Wendling F Bartolomei F Modolo J Neocortical/thalamic *in silico* models of seizures and epilepsy. In: Pitkänen A Buckmaster PS Galano poulou AS Moshé SL editors. Models of Seizures and Epilepsy. 2nd Ed. London: Academic Press (2017). p. 233–46.

[6] Löscher W. Critical review of current animal models of seizures and epilepsy used in the discovery and development of new antiepileptic drugs. Seizure. (2011) 20:359–68. 10.1016/j.seizure.2011.01.003 21292505

[7] Löscher W. Animal models of seizures and epilepsy: past, present, and future role for the discovery of antiseizure drugs. Neurochem Res. (2017) 42:1873–88. 10.1007/s11064-017-2222-z 28290134

[8] Muñoz LJ Carballosa-Gautam MM Yanowsky K García-Atarés N López DE. The genetic audiogenic seizure hamster from Salamanca: the GASH:Sal. Epilepsy Behav. (2017) 71:181–92. 10.1016/j.yebeh.2016.03.002 27072920

[9] Garcia-Cairasco N Umeoka EHL Cortes de Oliveira JA. The wistar audiogenic rat (WAR) strain and its contributions to epileptology and related comorbidities: history and perspectives. Epilepsy Behav. (2017) 71:250–73. 10.1016/j.yebeh.2017.04.001 28506440

[10] Poletaeva II Surina NM Kostina ZA Perepelkina O V Fedotova IB The krushinsky-molodkina rat strain: the study of audiogenic epilepsy for 65 years. Epilepsy Behav. (2017) 71:130–41. 10.1016/j.yebeh.2015.04.072 26228091

[11] Ribak CE. An abnormal GABAergic system in the inferior colliculus provides a basis for audiogenic seizures in genetically epilepsy-prone rats. Epilepsy Behav. (2017) 71:160–4. 10.1016/j.yebeh.2015.02.024 25812940 PMC4580487

[12] Terra VC Garcia-Cairasco N. NMDA-dependent audiogenic seizures are differentially inferior colliculus subnuclei. Behav Brain Res. (1994) 62:29–39. 10.1016/0166-4328(94)90035-3 7917031

[13] Garcia-Cairasco N Sabbatinit RM. Possible interaction between the inferior colliculus and the substantia nigra in audiogenic seizures in wistar rats. Physiol Behav. (1991) 50:421–7. 1745689 10.1016/0031-9384(91)90089-7

[14] Pinto HPP Carvalho VR Medeiros DC Almeida AFS Mendes EMAM Moraes MFD. Auditory processing assessment suggests that wistar audiogenic rat neural networks are prone to entrainment. Neuroscience. (2017) 347:48–56. 10.1016/j.neuroscience.2017.01.043 28188855

[15] Prieto-Martín AI Aroca-Aguilar JD Sánchez-Sánchez F Muñoz LJ López DE Escribano J. Molecular and neurochemical substrates of the audiogenic seizure strains: the GASH:Sal model. Epilepsy Behav. (2017) 71:218–25. 10.1016/j.yebeh.2015.05.025 26071997

[16] Drumond LE Kushmerick C Guidine PAM Doretto MC Moraes MFD Massensini AR. Reduced hippocampal GABAergic function in wistar audiogenic rats. Brazilian J Med Biol Res. (2011) 44:1054–9. 10.1590/S0100-879X2011007500118 21915472

[17] Mesquita F Aguiar JF Oliveira JA Garcia-Cairasco N Varanda WA. Electrophysiological properties of cultured hippocampal neurons from wistar audiogenic rats. Brain Res Bull. (2005) 65:177–83. 10.1016/j.brainresbull.2005.01.003 15763185

[18] López-López D Gómez-Nieto R Herrero-Turrión MJ García-Cairasco N Sánchez-Benito D. Overexpression of the immediate-early genes Egr1, Egr2, and Egr3 in two strains of rodents susceptible to audiogenic seizures. Epilepsy Behav. (2017) 71:226–37. 10.1016/j.yebeh.2015.12.020 26775236

[19] Doretto MC Fonseca CG Lôbo RB Terra VC Oliveira JAC Garcia-Cairasco N. Quantitative study of the response to genetic selection of the wistar audiogenic rat strain (WAR). Behav Genet. (2003) 33:33–42. 10.1023/a:1021099432759 12645820

[20] Sánchez-Benito D Gómez-Nieto R Hernández-Noriega S Murashima AAB de Oliveira JAC Garcia-Cairasco N. Morphofunctional alterations in the olivocochlear efferent system of the genetic audiogenic seizure-prone hamster GASH:Sal. Epilepsy Behav. (2017) 71:193–206. 10.1016/j.yebeh.2016.05.040 27492627

[21] Rossetti F Rodrigues MCA Oliveira JAC de Garcia-Cairasco N. EEG wavelet analyses of the striatum-substantia nigra pars reticulata-superior colliculus circuitry: audiogenic seizures and anticonvulsant drug administration in wistar audiogenic rats (war strain). Epilepsy Res. (2006) 72:192–208. 10.1016/j.eplepsyres.2006.08.001 17150334

[22] Garcia-cairasco N Doretto MC Ramalho MJ ANTUNES-RODRIGUESt J Antunes-rodrigues J. Audiogenic and audiogenic-like seizures: locus of induction and seizure severity determine postictal prolactin patterns. Pharmacol Biochem Behav. (1996) 53:503–10. 10.1016/0091-3057(95)02040-3 8866947

[23] Schmittgen TD Livak KJ Analyzing real-time PCR data by the comparative CT method. Nat Protoc. (2008) 3:1101–8. 10.1038/nprot.2008.73 18546601

[24] Gómez-Nieto R Rubio ME López DE. Cholinergic input from the ventral nucleus of the trapezoid body to cochlear root neurons in rats. J Comp Neurol. (2008) 506:452–68. 10.1002/cne.21554 18041785

[25] Damasceno S Menezes NB Rocha CS Matos AHB Vieira AS Moraes MFD. Transcriptome of the wistar audiogenic rat (WAR) strain following audiogenic seizures. Epilepsy Res. (2018) 147:22–31. 10.1016/j.eplepsyres.2018.08.010 30193173

[26] Yamagata K Kaufmann WE Lanahan A Papapavlou M Barnes CA Andreasson KI. Egr3/Pilot, a zinc finger transcription factor, is rapidly regulated by activity in brain neurons and colocalizes with Egr1/zif268. Learn Mem. (1994) 1:140–52. 10467592

[27] Michel MC Beck-Sickinger A Cox H Doods HN Herzog H Larhammar D. International union of pharmacology recommendations for the nomenclature of neuropeptide Y, peptide YY, and pancreatic polypeptide receptors. Pharmacol Rev. (1998) 50:143–50. 9549761

[28] Decressac M Barker RA. Neuropeptide Y and its role in CNS disease and repair. Exp Neurol. (2012) 238:265–72. 10.1016/j.expneurol.2012.09.004 23022456

[29] Yamazoe M Shiosaka S Emson PC Tohyama M. Distribution of neuropeptide Y in the lower brainstem: an immunohistochemical analysis. Brain Res. (1985) 335:109–20. 10.1016/0006-8993(85)90281-1 3891012

[30] Morris BJ. Neuronal localisation of neuropeptide Y gene expression in rat brain. J Comp Neurol. (1989) 290:358–68. 10.1002/cne.902900305 2592617

[31] Vezzani A Civenni G Rizzi M Monno A Messali S Samanin R. Enhanced neuropeptide Y release in the hippocampus is associated with chronic seizure susceptibility in kainic acid treated rats. Brain Res. (1994) 660:138–43. 10.1016/0006-8993(94)90847-8 7827990

[32] Vezzani A Sperk G Colmers WF. Neuropeptide Y : emerging evidence for a functional role in seizure modulation. Trends Neurosci. (1999) 22:25–30. 10.1016/S0166-2236(98)01284-3 10088996

[33] Vezzani A Sperk G. Overexpression of NPY and Y2 receptors in epileptic brain tissue: an endogenous neuroprotective mechanism in temporal lobe epilepsy? Neuropeptides. (2004) 38:245–52. 10.1016/j.npep.2004.05.004 15337376

[34] Stroud LM O'Brien TJ Jupp B Wallengren C Morris MJ. Neuropeptide Y suppresses absence seizures in a genetic rat model. Brain Res. (2005) 1033:151–6. 10.1016/j.brainres.2004.11.022 15694919

[35] Dubé C. Neuropeptide Y: potential role in recurrent developmental seizures. Peptides. (2007) 28:441–6. 10.1016/j.peptides.2006.08.034 17196709 PMC1852447

[36] Cardoso A Freitas-da-Costa P Carvalho LS Lukoyanov N V. Seizure-induced changes in neuropeptide Y-containing cortical neurons: potential role for seizure threshold and epileptogenesis. Epilepsy Behav. (2010) 19:559–67. 10.1016/j.yebeh.2010.09.008 20934916

[37] Deborah Lin EJ Young D Baer K Herzog H During MJ Differential actions of NPY on seizure modulation via Y1 and Y2 receptors: evidence from receptor knockout mice. Epilepsia. (2006) 47:773–80. 10.1111/j.1528-1167.2006.00500.x 16650144

[38] Vezzani A Rizzi M Conti M Samanin R. Modulatory role of neuropeptides in seizures induced in rats by stimulation of glutamate receptors. J Nutr. (2000) 130:1046S−8S. 10.1093/jn/130.4.1046S 10736379

[39] Gariboldi M Conti M Cavaleri D Samanin R Vezzani A. Anticonvulsant properties of BIBP3226, a non-peptide selective antagonist at neuropeptide Y Y1 receptors. Eur J Neurosci. (1998) 10:757–9. 10.1046/j.1460-9568.1998.00061.x 9749738

[40] Dumont Y Fournier A Quirion R. Expression and characterization of the neuropeptide Y Y5receptor subtype in the rat brain. J Neurosci. (1998) 18:5565–74. 10.1523/JNEUROSCI.18-15-05565.1998 9671648 PMC6793038

[41] Dumont Y Jacques D Bouchard P Quirion R. Species differences in the expression and distribution of the neuropeptide Y Y1, Y2, Y4, and Y5 receptors in rodents, guinea pig, and primates brains. J Comp Neurol. (1998) 402:372–84. 10.1002/(SICI)1096-9861(19981221)402:3<372::AID-CNE6>3.0.CO;2-2 9853905

[42] Kovac S Walker MC. Neuropeptides in epilepsy. Neuropeptides. (2013) 47:467–75. 10.1016/j.npep.2013.10.015 24210141

[43] Bosque JR Gómez-Nieto R Hormigo S Herrero-Turrión MJ Díaz-Casado E Sancho C Molecular tools for the characterization of seizure susceptibility in genetic rodent models of epilepsy. Epilepsy Behav. (2019) 1:106594 10.1016/j.yebeh.2019.106594 31685382

[44] Siderovski DP Willard FS. The GAPs, GEFs, and GDIs of heterotrimeric G-protein alpha subunits. Int J Biol Sci. (2005) 1:51–66. 10.7150/ijbs.1.51 15951850 PMC1142213

[45] Roman DL Traynor JR. Regulators of G protein signaling (RGS) proteins as drug targets: modulating g-protein-coupled receptor (GPCR) signal transduction. J Med Chem. (2011) 54:7433–40. 10.1021/jm101572n 21916427 PMC3208131

[46] Zmijewski JW Song L Jope RS Harkins L Cobbs CS. Oxidative stress and heat shock stimulate RGS2 expression in 1321N1 astrocytoma cells. Arch Biochem Biophys. (2001) 392:192–6. 10.1006/abbi.2001.2430 11488592

[47] Song L Jope RS. Cellular stress increases RGS2 mRNA and decreases RGS4 mRNA levels in SH-SY5Y cells. Neurosci Lett. (2006) 402:205–9. 10.1016/j.neulet.2006.03.023 16733081 PMC1618799

[48] Nunn C Zhao P Zou MX Summers K Guglielmo CG Chidiac P. Resistance to age-related, normal body weight gain in RGS2 deficient mice. Cell Signal. (2011) 23:1375–86. 10.1016/j.cellsig.2011.03.020 21447383

[49] Gold SJ Heifets BD Pudiak CM Potts BW Nestler EJ. Regulation of regulators of G protein signaling mRNA expression in rat brain by acute and chronic electroconvulsive seizures. J Neurochem. (2002) 82:828–38. 10.1046/j.1471-4159.2002.01002.x 12358788

[50] Mark MD Wittermann S Herlitze S. G protein modulation of recombinant P/Q-type calcium channels by regulators of G protein signalling proteins. J Physiol. (2000) 528:65–77. 10.1111/j.1469-7793.2000.00065.x 11018106 PMC2270115

[51] Eusemann TN Willmroth F Fiebich B Biber K Van Calker D. Adenosine receptors differentially regulate the expression of regulators of G-protein signalling (RGS) 2, 3 and 4 in astrocyte-like cells. PLoS ONE. (2015) 10:e0134934. 10.1371/journal.pone.0134934 26263491 PMC4532427

[52] Christensen KV Leffers H Watson WP Sánchez C Kallunki P Egebjerg J. Levetiracetam attenuates hippocampal expression of synaptic plasticity-related immediate early and late response genes in amygdala-kindled rats. BMC Neurosci. (2010) 11:9. 10.1186/1471-2202-11-9 20105316 PMC2848232

[53] Soprano DR Herbert J Soprano KJ Schon EA Goodman DS. Demonstration of transthyretin mRNA in the brain and other extrahepatic tissues in the rat. J Biol Chem. (1985) 260:11793–8. 4044580

[54] Blay P Nilsson C Owman C Aldred A Schreiber G. Transthyretin expression in the rat brain: effect of thyroid functional state and role in thyroxine transport. Brain Res. (1993) 632:114–20. 10.1016/0006-8993(93)91145-I 8149219

[55] Li X Masliah E Reixach N Buxbaum JN. Neuronal production of transthyretin in human and murine alzheimer's disease: is it protective? J Neurosci. (2011) 31:12483–90. 10.1523/JNEUROSCI.2417-11.2011 21880910 PMC3172869

[56] Zhou L Tang X Li X Bai Y Buxbaum JN Chen G Identification of transthyretin as a novel interacting partner for the δ subunit of GABA A receptors. PLoS ONE. (2019) 14:e0210094 10.1371/journal.pone.0210094 30615651 PMC6322723

[57] Sharma M Khan S Rahman S Singh LR. The extracellular protein, transthyretin is an oxidative stress biomarker. Front Physiol. (2019) 10:5. 10.3389/fphys.2019.00005 30733681 PMC6353848

[58] Talhada D Gonçalves I Reis Santos C Ruscher K. Transthyretin expression in the postischemic brain. PLoS ONE. (2019) 14:e0221555. 10.1371/journal.pone.0221555 31479465 PMC6719853

[59] Richardson SJ. Cell and molecular biology of transthyretin and thyroid hormones. Int Rev Cytol. (2007) 258:137–93. 10.1016/S0074-7696(07)58003-4 17338921

[60] Brett M Persey MR Reilly MM Revesz T Booth DR Booth SE. Transthyretin Leu12Pro is associated with systemic, neuropathic and leptomeningeal amyloidosis. Brain. (1999) 122:183–90. 10.1093/brain/122.2.183 10071047

[61] Jin K. Familial leptomeningeal amyloidosis with a transthyretin variant Asp18Gly representing repeated subarachnoid haemorrhages with superficial siderosis. J Neurol Neurosurg Psychiatry. (2004) 75:1463–1466. 10.1136/jnnp.2003.029942 15377697 PMC1738739

[62] Douglass C Suvarna K Reilly MM Hawkins PN Hadjivassiliou M. A novel amyloidogenic transthyretin variant, Gly53Ala, associated with intermittent headaches and ataxia. J Neurol Neurosurg Psychiatry. (2007) 78:193–5. 10.1136/jnnp.2006.093500 16971399 PMC2077663

[63] Ando Y Coelho T Berk JL Cruz MW Ericzon BG Ikeda SI. Guideline of transthyretin-related hereditary amyloidosis for clinicians. Orphanet J Rare Dis. (2013) 8:1–18. 10.1186/1750-1172-8-31 23425518 PMC3584981

[64] Ranganathan S Williams E Ganchev P Gopalakrishnan V Lacomis D Urbinelli L. Proteomic profiling of cerebrospinal fluid identifies biomarkers for amyotrophic lateral sclerosis. J Neurochem. (2005) 95:1461–71. 10.1111/j.1471-4159.2005.03478.x 16313519 PMC1540444

[65] Serot JM Christmann D Dubost T Couturier M. Cerebrospinal fluid transthyretin: aging and late onset alzheimer's disease. J Neurol Neurosurg Psychiatr. (1997) 63:506–8. 10.1136/jnnp.63.4.506 9343132 PMC2169793

[66] Biroccio A del Boccio P Panella M Bernardini S Di Ilio C Gambi D. Differential post-translational modifications of transthyretin in alzheimer's disease: a study of the cerebral spinal fluid. Proteomics. (2006) 6:2305–13. 10.1002/pmic.200500285 16552785

[67] Castaño EM Roher AE Esh CL Kokjohn TA Beach T. Comparative proteomics of cerebrospinal fluid in neuropathologically-confirmed alzheimer's disease and non-demented elderly subjects. Neurol Res. (2006) 28:155–63. 10.1179/016164106X98035 16551433

[68] Buxbaum JN Ye Z Reixach N Friske L Levy C Das P. Transthyretin protects alzheimer's mice from the behavioral and biochemical effects of A toxicity. Proc Natl Acad Sci USA. (2008) 105:2681–6. 10.1073/pnas.0712197105 18272491 PMC2268196

[69] Li X Buxbaum JN. Transthyretin and the brain re-visited: is neuronal synthesis of transthyretin protective in alzheimer's disease? Mol Neurodegener. (2011) 6:79. 10.1186/1750-1326-6-79 22112803 PMC3267701

[70] Silva CS Eira J Ribeiro CA Oliveira Â Sousa MM Cardoso I. Transthyretin neuroprotection in alzheimer's disease is dependent on proteolysis. Neurobiol Aging. (2017) 59:10–14. 10.1016/j.neurobiolaging.2017.07.002 28780366

[71] Liverman CS Cui L Yong C Choudhuri R Klein RM Welch KMA. Response of the brain to oligemia: gene expression, c-Fos, and Nrf2 localization. Mol Brain Res. (2004) 126:57–66. 10.1016/j.molbrainres.2004.02.028 15207916

[72] Stein TD Anders NJ DeCarli C Chan SL Mattson MP Johnson JA. Neutralization of transthyretin reverses the neuroprotective effects of secreted amyloid precursor protein (APP) in APPSw mice resulting in tau phosphorylation and loss of hippocampal neurons: support for the amyloid hypothesis. J Neurosci. (2004) 24:7707–17. 10.1523/JNEUROSCI.2211-04.2004 15342738 PMC6729623

[73] Kajiwara K Sunaga K Tsuda T Sugaya A Sugaya E Kimura M. Peony root extract upregulates transthyretin and phosphoglycerate mutase in mouse cobalt focus seizure. Biochem Biophys Res Commun. (2008) 371:375–9. 10.1016/j.bbrc.2008.04.094 18448067

[74] Cordon-Cardo C O'Brien JP Casals D Rittman-Grauer L Biedler JL Melamed MR. Multidrug-resistance gene (P-glycoprotein) is expressed by endothelial cells at blood-brain barrier sites. Proc Natl Acad Sci USA. (1989) 86:695–8. 10.1073/pnas.86.2.695 2563168 PMC286540

[75] Leveille-Webster CR Arias IM. The biology of the P-glycoproteins. J Membr Biol. (1995) 143:89–102. 10.1007/BF00234655 7731035

[76] Miller DS. Regulation of ABC transporters at the blood-brain barrier. Clin Pharmacol Ther. (2015) 97:395–403. 10.1002/cpt.64 25670036 PMC4363166

[77] Seegers U Potschka H Löscher W. Transient increase of P-glycoprotein expression in endothelium and parenchyma of limbic brain regions in the kainate model of temporal lobe epilepsy. Epilepsy Res. (2002) 51:257–68. 10.1016/S0920-1211(02)00156-0 12399076

[78] Lazarowski A Lubieniecki F Camarero S Pomata H Bartuluchi M Sevlever G. Multidrug resistance proteins in tuberous sclerosis and refractory epilepsy. Pediatr Neurol. (2004) 30:102–6. 10.1016/S0887-8994(03)00407-7 14984901

[79] van Vliet E Aronica E Redeker S Marchi N Rizzi M Vezzani A. Selective and persistent upregulation of mdr1b mRNA and P-glycoprotein in the parahippocampal cortex of chronic epileptic rats. Epilepsy Res. (2004) 60:203–13. 10.1016/j.eplepsyres.2004.06.005 15380564

[80] Liu X Yang Z Yang J Yang H. Increased P-glycoprotein expression and decreased phenobarbital distribution in the brain of pentylenetetrazole-kindled rats. Neuropharmacology. (2007) 53:657–63. 10.1016/j.neuropharm.2007.07.012 17845805

[81] Kwan P Sills GJ Butler E Gant TW Meldrum BS Brodie MJ. Regional expression of multidrug resistance genes in genetically epilepsy-prone rat brain after a single audiogenic seizure. Epilepsia. (2002) 43:1318–23. 10.1046/j.1528-1157.2002.156702.x 12423380

[82] Tishler DM Weinberg KI Hinton DR Barbaro N Annett GM Raffel C. MDR1 gene expression in brain of patients with medically intractable epilepsy. Epilepsia. (1995) 36:1–6. 10.1111/j.1528-1157.1995.tb01657.x 8001500

[83] Rizzi M Caccia S Guiso G Richichi C Gorter JA Aronica E. Limbic seizures induce P-glycoprotein in rodent brain: functional implications for pharmacoresistance. J Neurosci. (2002) 22:5833–9. 10.1523/JNEUROSCI.22-14-05833.2002 12122045 PMC6757954

[84] van Vliet EA van Schaik R Edelbroek PM Voskuyl RA Redeker S Aronica E. Region-specific overexpression of P-glycoprotein at the blood-brain barrier affects brain uptake of phenytoin in epileptic rats. J Pharmacol Exp Ther. (2007) 322:141–7. 10.1124/jpet.107.121178 17392402

